# Supplementary material for: Aggregation Characteristics of Tau Phosphorylated by Various Kinases as Observed by Quantum Dot Fluorescence Imaging
Source: Int J Mol Sci. 2025 Oct 17;26(20):10122. doi: 10.3390/ijms262010122 (PMC12563518; doi:10.3390/ijms262010122)
Supplement: Supplementary file 1 [file ijms-26-10122-s001.zip › ijms-3913277-supplementary.pdf]

International Journal of
Molecular Sciences

MDPI

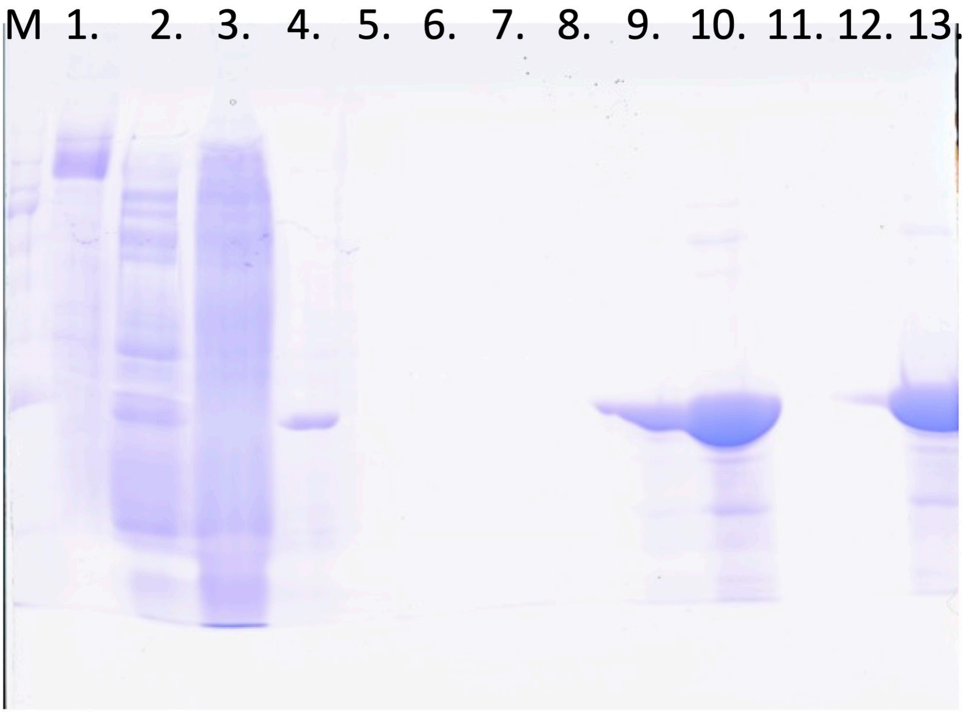

Supplementary Figure S1. SDS-PAGE gel image of samples obtained during mouse MBD tau purification. (M) Marker. (1) Soluble fraction after sonication. (2) Precipitate after sonication. (3) Precipitate after heat treatment. (4) Soluble fraction after heat treatment. (5) Filtrate waste liquid obtained when applying the post-heat treatment soluble fraction to the UNOsphere™ S column. (6) Filtrate waste liquid obtained when washing tau adsorbed onto the UNOsphere™ S column. (7) Filtrate waste liquid obtained when tau eluted from the UNOsphere™ S column was applied to a TOYOPEARL® Butyl-650 column. (8) Filtrate waste liquid obtained when washing tau adsorbed onto the TOYOPEARL® Butyl-650 column. (9) Tau solution eluted from the TOYOPEARL® Butyl-650 column. (10) Tau solution obtained by centrifugal concentration. (11) First dialysate. (12) Dialysate after overnight incubation. (13) Final tau solution.

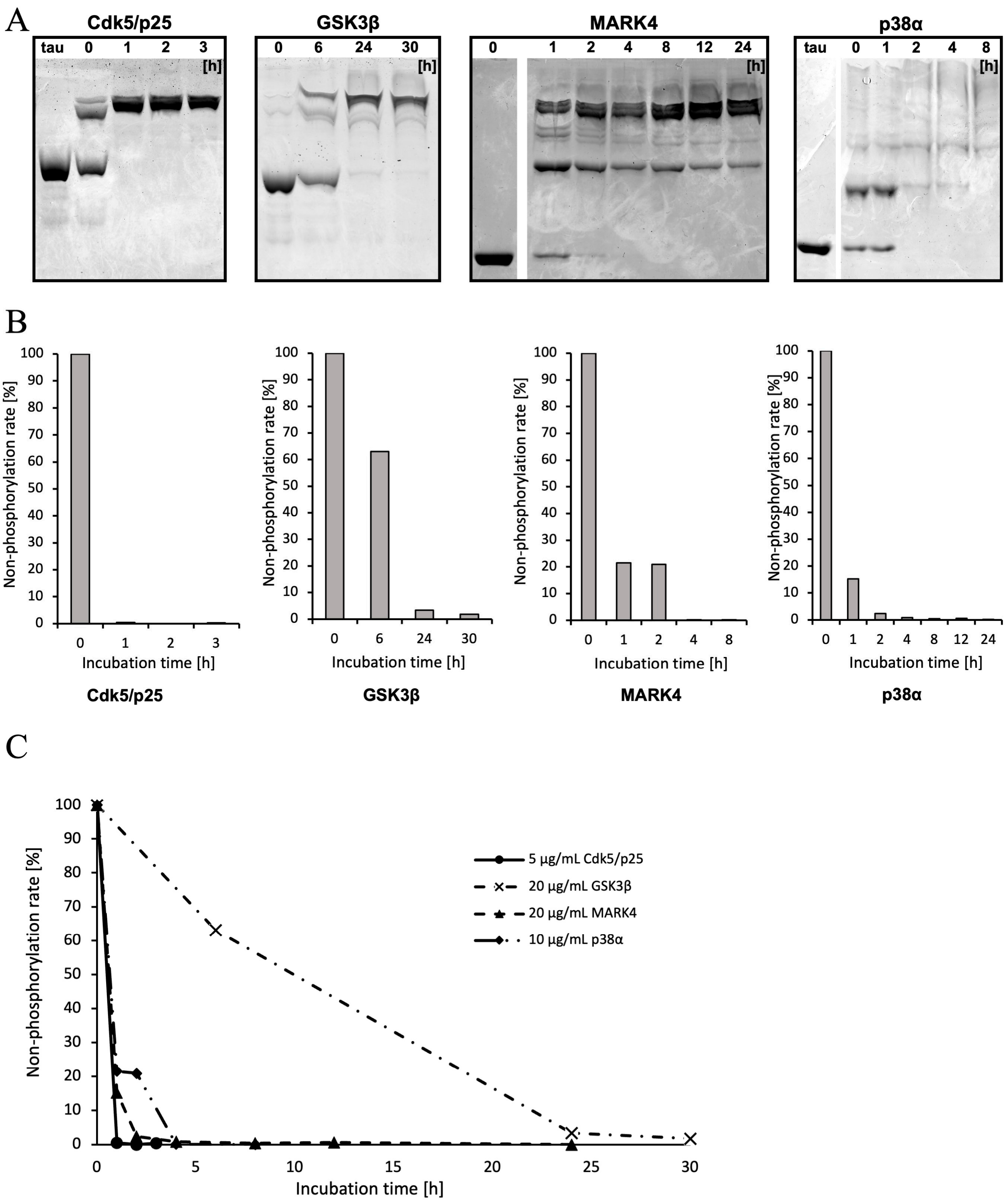

Supplementary Figure S2. Confirmation of phosphorylation saturation of mouse MBD tau by Phos-tag SDS-PAGE. (A) Phos-tag SDS-PAGE gel. (B) Quantitative results of bands obtained from the Phos-tag SDS-PAGE gel using ImageJ software. The intensity of the band for unphosphorylated tau was set to 100%, and the relative intensity of the band at each reaction time was calculated. (C) Line graph of the quantitative results calculated in panel (B).

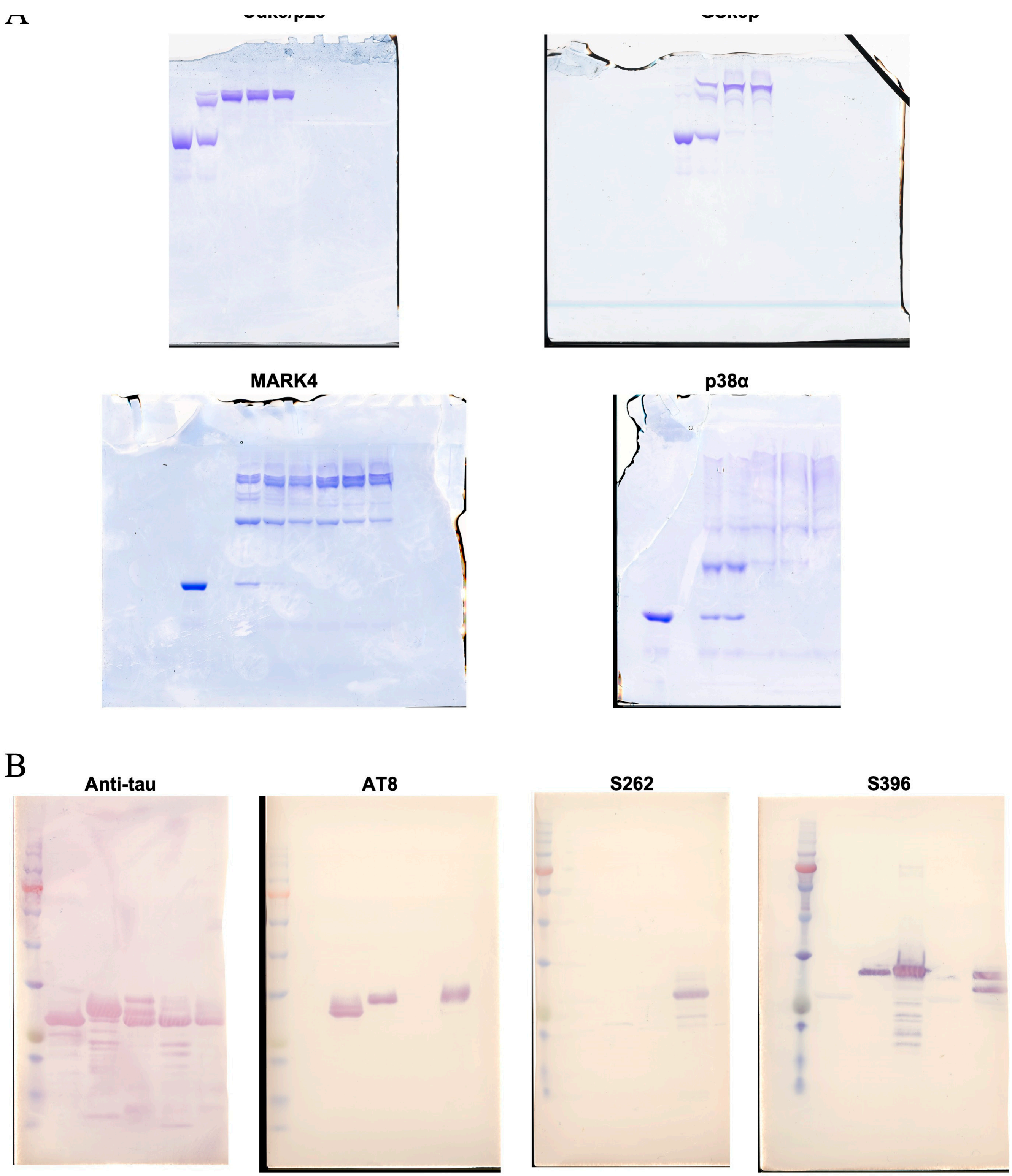

Supplementary Figure S3. Original photographs of Phos-tag SDS-PAGE gel and Western blot membrane. (A) Original photographs of Phos-tag SDS-PAGE gel. (B) Original photographs of Western blot membrane.

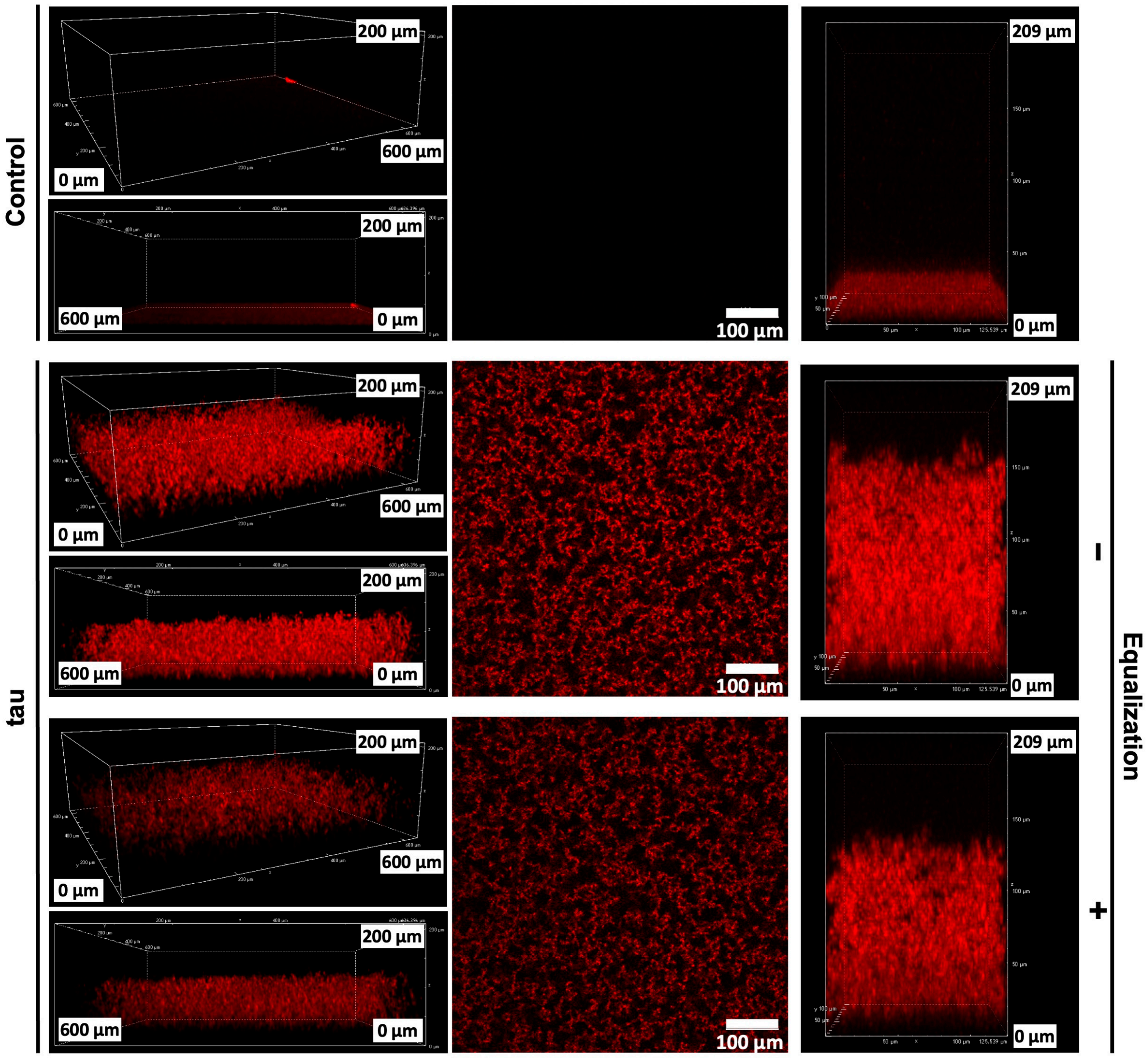

Supplementary Figure S4. The original 3D reconstructed image used in Figure 2 and its slice images. The right side shows a side view of an enlarged image of the 3D reconstructed image.

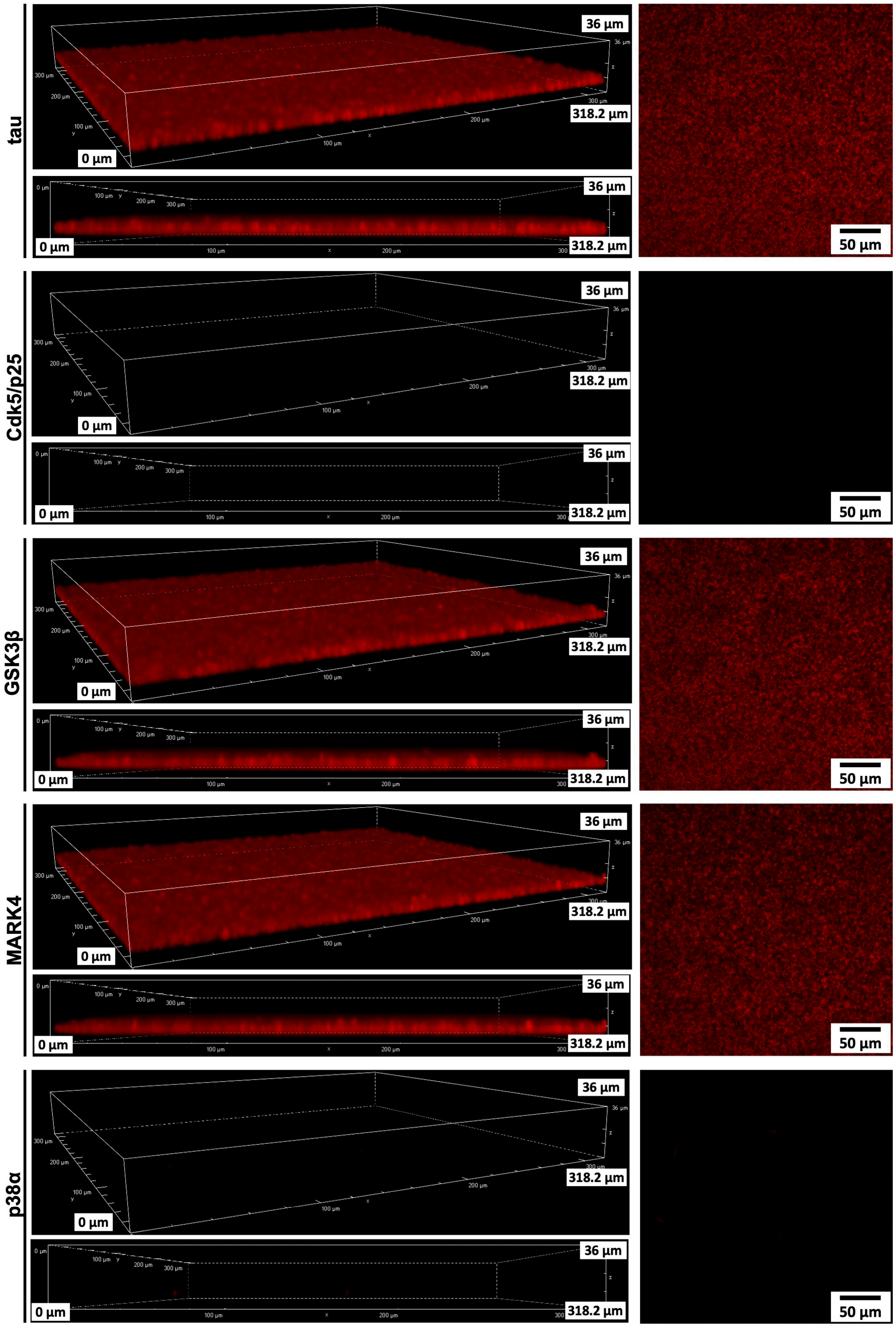

Supplementary Figure S5. The original 3D reconstructed image used in Figure 4 and its slice images.

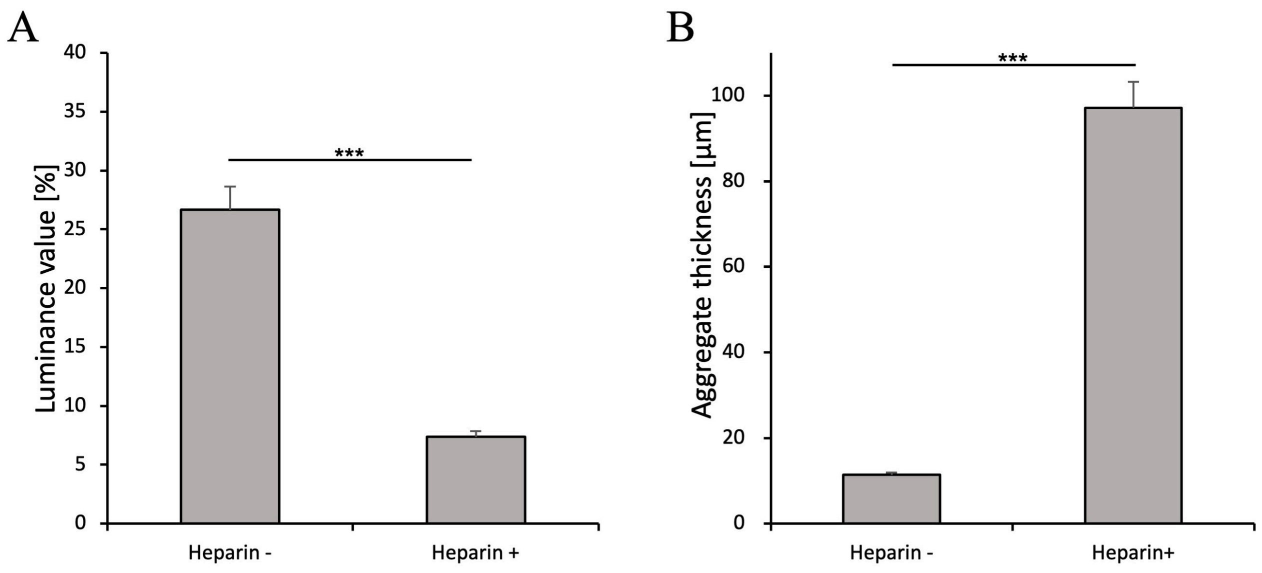

Supplementary Figure S6. Comparison of non-phosphorylated tau aggregation with and without heparin. (A) The right side shows a side view of an enlarged image of the 3D reconstructed image. Aggregate density quantified from slice images. Aggregate density was quantified using ImageJ software. Data represent the mean; error bars are derived from images obtained by dividing a single slice image into four sections. Statistical analysis was performed using *t*-tests. *** p<0.001 (n = 4). (B) Aggregate thickness quantified from the side view. Aggregate thickness was quantified using ImageJ software. Data represent the mean; error bars are derived from three points: left, center, and right of the side view. Statistical analysis was performed using *t*-tests. *** p<0.001 (n = 3).
